# Cramps

**Published:** 1859-10

**Authors:** 


### CRAMPS.

It was in early May, our first night out from New Orleans, a thunderstorm came up suddenly. We were just sitting down to the supper-table, all hearts seemed glad at soon entering on that series of gratifications which attends the usual summer tour north. Out of doors it was as dark as midnight; the rain fell in torrents; the angry hustlings of the wind, as it swept over the boiling eddies, the rattling of the machinery, the sharp, quick puffing of the steam-pipe showing that the engine was on its highest strain; the muttering of the deep thunders, with flashes of lightning so keen and vivid, that the splendid astrals of the cabin seemed to give no light at all, these, together, were not half so disquieting as certain yells of human agony, which came up from the deck below. Only ourself knew the cause, for we had spent the previous hour or two among the already-dying from the sudden and fearful onslaught of "Cholera." It was the terrible "*cramp*," which attends certain phases of that disease, that caused those shrieks which rose above the din of machinery, wind, and thunder.

What is this "*cramp*" of cholera, or colic, or some other form of disease, coming on as suddenly as the lightning, and going as suddenly away ; but, during its presence, is like the tearing out of the heart strings? It arises from the veins being so full of blood that they swell out, press against the large nerves, and thus impede the circulation of that all-important vital agency to a great extent, or in smaller nerves it neuralgia, which is literally "nerve-ache."

What causes that unusual fulness of the veins? The blood is so impure, so thick, so full of disease, that it cannot flow along by nature's ordinary agencies ; in proportion as it is thick, it is cold, and the man is cold, as witness the death-damps of the dying. Thus it is, that during epidemic cholera the blood of persons is so thick and cold for days and weeks before an attack, that the pulse, which, in health, as to grown persons, beats from sixty-six to seven-two times in a minute, now strikes so laboriously, so feebly, and so slow, that it counts but fifty, and sometimes but forty-two.

The above narration has been given to impress a practical principle of action on the memory. When a person is attacked with cramp, get some hot water quietly and expeditiously (for noise and exclamations of grief and alarm still further disturb the nervous equilibrium), put the sufferer in the water as completely as possible ; and thus heat is imparted to the blood, which sends it coursing along the veins, and the pain is gone. While the water is in preparation, rub the cramped part very briskly with the hand or a woolen flannel, with your mouth shut. But why keep the mouth shut? You can rub harder, faster, and more efficiently, besides it saves the sufferer from meaningless and agonizing inquiries. A man in pain does not want to be talked to—he wants relief, not words. If all could know, as physicians do, the inestimable value of quiet composure, and a confident air on the part of one who attempts to aid a sufferer, it would be practised with ceaseless assiduity by the considerate and the humane.

